# Photodynamic Therapy in the Inactivation of Microorganisms

**DOI:** 10.3390/antibiotics9040138

**Published:** 2020-03-25

**Authors:** Adelaide Almeida

**Affiliations:** Biology Department and CESAM, University of Aveiro, 3810-193 Aveiro, Portugal; aalmeida@ua.pt; Tel.: +351-234-370-784

The growing emergence of microbial resistance to conventional antimicrobials, due their dissemination in the environment, and excessive or inadequate prescriptions, associated with the globalization of pathogenic microorganisms’ transmission, make the discovery of new effective therapies to combat infection of extreme urgency. It was estimated that, if nothing will be done in the meantime, the cost of microbial resistance in terms of global production lost between 2015 and 2050 would be 100 trillion USD, and by 2050 microbial resistance will kill 10 million people per year, outweighing the death caused by cancer [1].

As the development of new conventional antimicrobials is unlikely to solve the problem of microbial resistance, it is a matter of time until microorganisms develop resistance to the new drugs [2]. So, the new alternative strategies must have a different mechanism(s) of action than conventional antimicrobials. Antimicrobial photodynamic therapy (aPDT) seems to be a very promising alternative to conventional antimicrobials to be used not only in human medicine, but also in other areas, such as in veterinary medicine, agro-food areas and wastewater treatment [3,4,5,6,7,8].

aPDT has already demonstrated its effectiveness against a wide range of microorganisms like Gram-positive and Gram-negative bacteria, viruses, fungi and parasites [9,10,11,12,13,14,15,16], independently of their resistance to conventional treatment [17,18,19]. This approach requires the presence of a photosensitizer (PS), light and oxygen. The PS when excited by light in the presence of dioxygen produces reactive oxygen species (ROS), such as superoxide radical anions, hydrogen peroxide and hydroxyl radicals (by the type I mechanism), or singlet oxygen, ^1^O_2_, (by the type II mechanism) [20,21]. These highly cytotoxic species are extremely reactive and strongly interact, at the same time, with a variety of vital biomolecules, mainly lipids, proteins and nucleic acids, leading to irreversible and rapid microbial inactivation [22,23].

A great advantage of aPDT is its multi-target mechanism, which makes it highly unlikely that microorganisms develop resistance, contrarily to what happens with conventional antimicrobials that generally work on a one-target principle [9,23,24,25,26]. Despite the aPDT advantages and its efficacy, there is still room for new improvements in order to transpose this technology to practice.

Some important aspects to be considered in this battle are related to the development of synthetic routes to produce new cost-effective and efficient PSs and to design new photodynamic protocols where the amount of PS required and/or the treatment time is reduced. It is recognized that an easy preparation of a PS associated with a low price are important features to take into account. Nevertheless, it is difficult to obtain a broad spectrum-of-action PS that fulfill these characteristics. In general, although the synthetic procedure is simple, the separation and purification processes are laborious, time-consuming and costly [27]. In fact, during the past two decades, many highly efficient PSs have been produced, but the chances to reach the market remain extremely low, not only due to the existing approval framework but also due to the laborious and expensive processes involved in their preparation and purification. Recently a PS formulation (FORM), based on a non-separated mixture of five cationic *meso*-tetraarylporphyrins, was equally effective in the photoinactivation of Gram-negative and Gram-positive bacteria, fungi, viruses and biofilms when compared with the highly efficient 5,10,15-tris(1-methylpyridinium-4-yl)-20-(pentafluorophenyl)porphyrin tri-iodide (Tri-Py(+)-Me), one of the constituents of FORM [27,28,29]. The effective viable-cell reduction of Gram-positive and Gram-negative bacteria with FORM provided promising indications toward its use, which would lead to a substantial decrease in costs and production time, paving its potential to field exploitation. Additionally, the combination of PS with different adjuvants like the inorganic salts sodium bromide, sodium azide, sodium thiocyanate and potassium iodide (KI) demonstrated a potentiate effect in the aPDT efficiency [29,30,31,32,33]. For example, the combination of different PSs with KI allowed a reduction in the PS concentration up to 1000 times, even against biofilms which are not so prone to inactivation with a PS when used alone [29]. 

Other aspect to be considered when establishing an optimal aPDT protocol is the PS mechanism of action [20,21]. It is important to evaluate the type of ROS produced during aPDT in order to determine the suitable conditions in which microbial photosensitization should operate and to design improved PS molecules. Although it is well established that the efficiency of aPDT is related to the ability of a PS to generate ^1^O_2_ (type II mechanism) and/or free radical species (type I mechanism) in the course of the photodynamic process [20,21], the possibility of oxygen-independent photoinactivation leading to the killing of pathogenic microorganisms, which may be termed the “Type III photochemical pathway”, has also been suggested [34]. The proposed mechanism involves photoinduced electron transfer that produces reactive inorganic radicals, which might be useful to treat anaerobic infections or infections in hypoxic tissues [34].

The development of light sources optimized for the antimicrobial application is another aspect that should be addressed. To overcome the limitations of lasers and non-coherent light sources, more economic, homogenous and powerful arrays based on light emitting diodes (LEDs) are needed [35]. Beside the light source, light irradiance and total light dose play an important role in the effectiveness of the aPDT and should always be considered when establishing an optimal antimicrobial protocol [36]. Considering the same light source and a fixed light dose applied at different light irradiances, phage inactivation was significantly higher when low light irradiances were used [36]. These LED systems can also be used for obtaining information on responsible endogenous PSs. Microbial inactivation based on endogenous PSs is, at present, attracting increasing attention by the scientific community due to its intrinsic antimicrobial effect without the addition of exogenous PSs [8,37,38,39]. The applicability of endogenous PSs for aPDT, as already observed when blue light therapy (aBLT) protocols are used, reduces the possibility of potential harmful effects on animal cells and also the impact in non-pathogenic microorganisms [8,39].

In consideration of so many new aspects related to the optimal photodynamic approach in the inactivation of microorganisms, it has been my pleasure to edit a joint presentation of the results from different research groups in one special scientific publication challenging researchers to respond to the currently underutilization in clinic and environmental applications. Besides, it is essential to raise awareness among health authorities and policy makers about the very serious emerging problem of microbial resistance. It is necessary to increase the current economical efforts (investment) for the discovery of new antimicrobial drugs, but it is also essential to develop new alternative approaches to conventional antimicrobials, such as aPDT. Otherwise, if nothing is done in the meantime, we can go back to the pre-antibiotic era, and not only the treatment of infectious diseases would be affected, but also several common clinical procedures, such as cesarean sections, organ transplants and chemotherapy, which strictly depend on the use of traditional antimicrobials to prevent infections, may be at risk.
